# *‘Moving on and feeling good’:* a feasibility study to explore the lifestyle behaviours of young adults with intellectual disabilities as they transition from school to adulthood—a study protocol

**DOI:** 10.1186/s40814-015-0044-9

**Published:** 2016-01-29

**Authors:** Fiona Mitchell, Andrew Jahoda, Catherine Hankey, Lynsay Matthews, Heather Murray, Craig Melville

**Affiliations:** 1 Institute of Health and Wellbeing, University of Glasgow, Glasgow, Scotland UK; 2 MRC/CSO Social and Public Health Sciences Unit, Institute of Health and Wellbeing, University of Glasgow, Glasgow, Scotland UK; 3 Institute of Health and Wellbeing, Robertson Centre for Biostatistics, University of Glasgow, Glasgow, Scotland UK

**Keywords:** Intellectual disabilities, Lifestyle behaviours, Physical activity, Diet, Transition, Weight gain prevention

## Abstract

**Background:**

The transition from adolescence to adulthood is a ‘high-risk’ period for weight gain in the general population. There is speculation that this may also be a risk period for adults with intellectual disabilities; however, there has been no research which has monitored change in health indicators. Since adults with intellectual disabilities have higher rates of obesity and engage in more sedentary behaviour and less physical activity than the general population, there is a need to understand more about the lifestyle behaviours of this population during the transition to adulthood. This protocol paper will provide details of the *moving on and feeling good* feasibility study, designed for young people with intellectual disabilities.

**Methods/design:**

A multi-point recruitment strategy will be used to recruit 30 participants with a mild-moderate level of intellectual disability. The aim of the feasibility study is to examine the feasibility of recruitment, participant retention and the measurement of relevant health behaviour outcomes. The study will assess the feasibility of monitoring weight, diet and physical activity levels in adolescents over a 12-month transitional period from school to adult life. This mixed method study will provide insight into the lives of young people with intellectual disabilities and will examine the use of Walker et al.’s social-ecological approach to promote self-determination specific to lifestyle behaviours, during this transition period. Baseline data will be collected during the final year of school, with follow-up data collection at 6 and 12 months. Anthropometric (weight, height, waist and hip circumference), objective physical activity measures (7-day accelerometer wear) and dietary and choice measures will be collected at each time point to assess the feasibility of measuring diet patterns, food frequency, physical activity and BMI. Furthermore, ten participants will be selected for short semi-structured scoping interviews at baseline and 12-month follow-up, to gain information on psychological, social and environmental factors which might affect behaviour change.

**Discussion:**

The outcomes from the feasibility study will aid the development and piloting of a sufficiently powered randomised controlled trial. This would allow us to evaluate the effectiveness and sustainability of a lifestyle behaviour intervention, over a 5-year transition period.

**Electronic supplementary material:**

The online version of this article (doi:10.1186/s40814-015-0044-9) contains supplementary material, which is available to authorized users.

## Background

Obesity is a major public health concern internationally [1], and there is clear evidence of the negative impact of obesity on health, with an increased risk of chronic health problems [2] and increased mortality [3]. The prevalence of obesity in adults with intellectual disabilities is significantly higher than in the general population [4, 5]. Research also suggests cardio-metabolic risk factors are prevalent in individuals with intellectual disabilities from adolescence [6], contributing to the higher disease burden for individuals with disabilities [7]. Therefore, the need to support adults with intellectual disabilities to make positive lifestyle behaviour changes, as a means to health improvement, has been recognised globally [8–11].

There is also evidence that suggests young people with intellectual disabilities have higher rates of obesity than typically developing young people. For example, a population-based study indicated that the prevalence of obesity in young adults with intellectual disabilities aged 16–24 was 28.1 %, compared to 10.5 % in a comparison sample of young adults, aged 16–24, who did not have intellectual disabilities (odds ratio = 3.37, 95 % CI 2.12, 5.37; Melville et al. [4]). The comparison BMI data used was from participants in a nationally representative household survey aged 16–24 and living in the same geographical area as the young adults with intellectual disabilities. However, the study did not examine whether the large difference in the obesity rates could be explained by differences in the samples, such as gender or socio-economic status. The prevention of obesity is a priority for health care and highlighted as important for young adults with intellectual disabilities internationally [8, 10, 12, 13].

Research suggests a range of factors may contribute to increased obesity rates, such as inherent biological and psychological vulnerabilities. For example, adults with intellectual disabilities have higher levels of depression than individuals without an intellectual disability (3.8 % [14] compared to 2.6 % [15]) and lower levels of health literacy. In addition, there is also evidence of social inequalities, such as economic disadvantage (e.g. greater material hardship, living in more deprived neighbourhoods, reduced community and social participation) [16]. While there is a growing body of research which suggests that adults with intellectual disabilities lead more sedentary and less physically active lifestyles than the general population [17–21], there is limited evidence about the lifestyle behaviours of young people with intellectual disabilities. Population-based studies from the general population suggests that the 5-year transitional period between adolescence and young adulthood is known to be a period of increased risk for the development of obesity [22], unhealthy diet and low physical activity levels [23]. Transitioning from school to adulthood may mean a young person is leaving behind a structured supportive environment for one that is less so. For young people with intellectual disabilities, this change in environment is likely to be more pronounced, with research suggesting up to 75 % adults with intellectual disabilities are underemployed or unemployed up to 3 years after leaving school [24]. This may result in more time spent sedentary at home, or in a residential setting. A recent systematic review highlights there has been no research to date which has monitored change in weight, or lifestyle behaviours relevant to health with this population [25]; it seems likely this transition would be a high-risk period for young people with intellectual disabilities to experience weight gain.

Peers are an important part of young people’s lives [26]; however, there are likely to be fewer opportunities for adults with intellectual disabilities to socialise in adulthood [27] which may also impact on opportunities to participate in physical activity or sport. In addition, families and carers play an important role in the lifestyle behaviours of an individual’s intellectual disabilities. Research has suggested that parents and carers’ own lack of motivation or time to be active can be a barrier to supporting an individual with intellectual disabilities to participate in physical activity [28]. Further, recent research [29] suggests that some paid carers and family members who were supporting individuals with intellectual disabilities to take part in a walking intervention rewarded walking and good behaviour with sweet treats and fizzy drinks. Therefore, the positive and negative influence that parents and carers’ have on an individual’s choice about (or lack of) diet and activity needs further examination.

A growing international literature base suggests that individuals with intellectual disabilities have low levels of self-determination [30–33]. As such, researchers have emphasised the need to consider how the environmental context of the lives of individuals with intellectual disabilities impacts on their self-determination [34, 35]. For example, a reluctance to tackle tasks independently means that parental support may have a positive impact on a young person’s confidence about participating in physical activities. Previous studies by the authors have also highlighted the need to take into account the context of the lives of people with intellectual disabilities when applying models of healthy lifestyle behaviours [36, 37]. Adding environmental mediators (e.g. the influence of the family, peers, school culture, work/college culture, area lived in, community, green space, opportunities for PA, autonomy over dietary and PA behaviours) to a self-determination model offers a more sophisticated theoretical framework for future weight gain prevention research. A key element of this feasibility study is therefore to identify relevant environmental factors acting as potential mediators on self-determination that inform our understanding of lifestyle behaviours and health, at the time of transition.

**Fig. 1:**
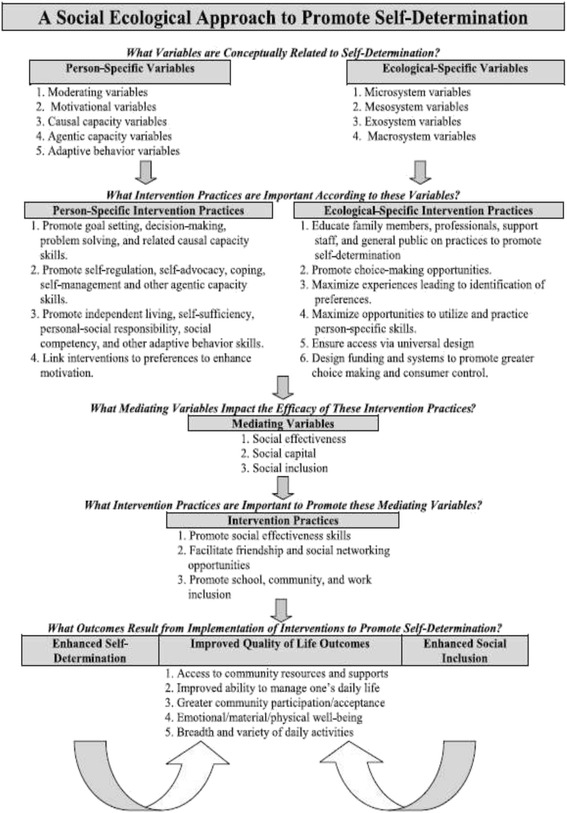
Data collection points of feasibility study

The health and wellbeing of young people with intellectual disabilities is an area of concern, and tackling the rising rates of obesity before adulthood could prevent the development of obesity-related diseases. Currently, there are no published intervention studies which aim to improve the lifestyle behaviours, health and wellbeing of young people with intellectual disabilities, as they transition into adulthood. This feasibility study is the first part of a larger body of work which will explore and monitor the lifestyle behaviours of young adults with intellectual disabilities, over the transition from school to adulthood. The aim of the feasibility study is to examine the feasibility of recruitment, participant retention and the measurement of relevant health behaviour outcomes. In addition, the work will explore the feasibility of applying an existing socio-ecological model of self-determination [35] specifically to lifestyle behaviours, over this transition period (see Fig. 1). This model identifies ‘person-specific’ and ‘ecological-specific’ variables that can affect levels of self-determination in this population. It also identifies ‘mediating’ variables which could impact on the efficacy of interventions, which promote self-determination. This feasibility study will explore the significance of these mediators in young people with intellectual disabilities’ lives by exploring levels of social effectiveness (friendships, recruiting social support networks, joining groups, managing one’s life and daily routines, etc.), social capital (networks of social ties, supports, relationships, trust, cooperation, affiliations) and social inclusion (societal acceptance of people with disabilities within school, work and community settings). Understanding more about these mediators could then be used to inform an intervention to promote self-determination for lifestyle behaviours in young adults with intellectual disabilities.

The term transition is used variably, with some authors [38] suggesting it is a gradual process spanning adolescence, while others suggesting it has more to do with a specific transition in one’s life, e.g. from school to adolescence [39]. In this study, we are interested specifically in the change in the physical environment from school to work or adult life. This study will monitor participants over a period of 12 months, which will allow us to assess the feasibility of retaining participating in a study over this transition period. It is out with the scope of this small-scale study to follow up over a longer transition period. The outcomes from the feasibility study will aid the development and piloting of a sufficiently powered randomised controlled trial. This would allow us to evaluate the effectiveness and sustainability of a lifestyle behaviour intervention, over a 5-year transition period.

The Medical Research Council (MRC) guidelines for evaluating complex interventions [40] will be used as the basis for the body of work described above. In keeping with phases one and two of the MRC framework, this study proposes to examine the feasibility of recruitment, participant retention and the measurement of relevant outcomes. In addition, it will explore the applicability of focussing on self-determination constructs for a future intervention for the prevention of weight gain. The study will address the following research questions:

1. What are the most effective routes for recruiting young adults with intellectual disabilities?
2. What are the recruitment and retention rates during a 12-month longitudinal study of young adults with intellectual disabilities?
3. Can lifestyle behaviour outcomes (weight, diet, physical activity) and self-determination be followed up over time and are any changes observed?
4. What are the environmental mediators of self-determination that need to be considered in relation to lifestyle behaviours and health, at the time of transition?
5. Are Dietary Instrument for Nutrition Education (DINE) and multiple recall questionnaires appropriate dietary measures for young adults with intellectual disabilities?

## Methods/design

**Fig. 2:**
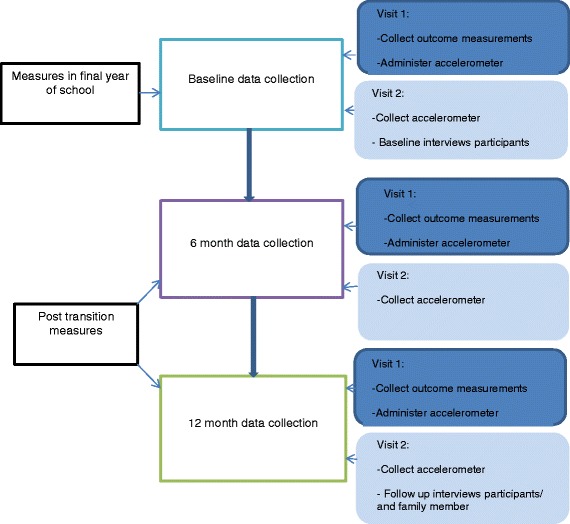
Socio-ecological model of self-determination

Outcomes from a 12-month longitudinal data will address questions 1–3 (Fig. 2). To answer research question 4, a sub-sample of participants will be included in a nested study involving semi-structured interviews before and after leaving school. In addition, follow-up interviews with a sample of family members will be carried out at the 12-month follow-up visit.

### Inclusion criteria

Participants will be invited to join the study if they are over 16 years of age and have a medical diagnosis of mild-moderate intellectual disability (individuals who have specific disorders of developmental delay, e.g. speech and language delay or dyslexia or autism spectrum disorder without an intellectual disability, will not be included in the study). In addition, individuals will be included if they are in their final year of school and are independently ambulatory. The researcher will ask the teachers to identify students who fit these criteria. The researcher will also send out information to parents which states the inclusion criteria for taking part in the study. Parents are asked to contact the researcher if they do not think their child meets the inclusion criteria. Individuals will be invited to take part in the research, following the procedure described below. Ethical approval has been granted by the University of Glasgow ethics committee.

### Recruitment procedure

Thirty young adults with intellectual disabilities will be invited to participate from several recruitment points. The proposed number of participants is based on recommendations from authors specialising in feasibility and pilot studies [41, 42]. The sample will introduce sufficient variance to examine the feasibility of a larger study and allow the identification of potential environmental mediators.

Researchers have identified the need for a recruitment strategy [36, 43, 44]. A strategy has been designed (Additional file 1) to guide the recruitment process, based on the framework provided by Foster et al. [43]. Recruitment will be carried out from December 2014 to June 2015. The authors have strong links with mainstream and additional support needs (ASN) schools and community groups for disabled people. ASN schools will be contacted first as the authors expect this will be the most successful recruitment point, based on the number of students who will meet the inclusion criteria. If the recruitment target of 30 pupils from ASN schools is not met (schools which are within 20 miles of a university), mainstream schools will then be targeted, followed by disability clubs.

Potential participants will be identified from these three sources and recruitment rates monitored to identify the most successful recruitment methods that could inform a future large-scale trial. If young people are interested in the study (based on an overview of the study provided by the researcher, teachers/club leader), they will be given an information pack which contains details of the study. Participants can signal interest in the study by informing the researcher verbally, or signing and returning a tear-off slip in the information pack and returning it to their teacher/club leader. The information sheets have been designed and piloted for individuals with mild-moderate intellectual disabilities. The researcher will also offer to read information sheets aloud. Schools/clubs will then be contacted to arrange a consent and baseline data visit.

The researcher will ensure all the participants have capacity to consent to the research by asking a variety of questions to ascertain that the participant understands what is being asked of them (e.g. Can you tell me what this study is about? Do you know what this belt (accelerometer) is for? Who decides if you want to take part? What do you do if you do not want to take part anymore?). If the researcher does not believe a participant fully understands the study, they will not be included in the study.

The researcher working with the young people is an experienced health psychologist who has worked with individuals with a range of intellectual disabilities. Therefore, she will ensure that the participants understand what is being asked of them, have the opportunity to ask any questions and can change their mind about participation at any point. The researcher will also ensure she is communicating clearly, using appropriate language for the level of intellectual disability and is patient and empathic in her interactions with the young people.

### Outcome measures

The following assessments will be completed by participants at baseline, 6- and 12-month follow-up.

#### Anthropometric measurements

All anthropometric measurements will be made using standard procedures in accordance with the International Standards for Anthropometric Assessment [45] with the participant wearing light clothes and without shoes. All measurements will be made in duplicate and a final mean value calculated. Weight in kilograms (kg) will be measured to the nearest 100 g, using SECA 877 scales (SE approval class III; SEA Germany). Height in metres (m) will be measured to the nearest 1 mm using the SECA Leicester stadiometer (SECA, Germany). The height (m) and weight (kg) will be used to calculate body mass index (BMI) using the formula: BMI = weight/height^2^ (kg/m^2^).

Waist and hip circumference will be measured to the nearest 0.5 cm. Waist circumference will be measured at the midpoint between the iliac crest and the lowest rib, in full expiration while the participant is standing. The hip circumference will be measured at the widest part of the hip including the gluteal muscles.

#### Dietary habits

The feasibility of measuring dietary habits will be assessed using two different methods. Usual dietary patterns will be captured in interviews with participants using the ‘DINE’ food frequency questionnaire [46]. The DINE questionnaire is designed to make a quick initial assessment of the amount of total fat and dietary fibre in an individual’s usual diet. The questionnaire lists 19 foods or food groups which have been pre-scored according to the relative amount of fat or fibre contained in an average portion of the food. These scores are then weighted by the daily or weekly frequency of intake reported by the respondent and summed to give overall fat and fibre scores. This allows the interviewer to categorise a diet immediately into low-, medium- or high-fat and fibre intake. An additional question provides an index of the balance of saturated and unsaturated fat in the diet. This questionnaire can be scored by either the participant or interviewer. For this population group, the interviewer will read the questions aloud to the participant then will score each food group for them and provide feedback on their scores. The DINE method can be used by interviewers without nutrition expertise to identify problem food groups and to direct discussion about beneficial dietary changes.

Patterns of diet across the study will also be estimated using the 24-h recall method. This method requires an interviewer to ask the participant to remember in detail all the food and drink they consumed during the past 24 h. The interviewer will write down the respondent’s answers. As a retrospective method, it relies on an accurate memory of intake, reliability of the respondent not to under-/misreport and an ability to estimate portion size. While previous studies have explored the dietary behaviours in individuals with intellectual disabilities, this information is often obtained through paid carers [17]. Changing shifts and care which is not 24 h could pose problems with the reliability of this method. Where possible, individuals with intellectual disabilities own responses and views should be sought. While there may be an argument that those with more severe-profound intellectual disabilities may not be able to articulate a reliable account of what has been consumed in a questionnaire/interview format, this may not be true for those with mild-moderate intellectual disabilities. Currently, there is no published research which has explored the feasibility of using these dietary measures with individuals with mild-moderate intellectual disabilities; therefore, an important component of this feasibility study is to examine the reliability and validity of these measures. The individual interviews will also provide contextual information concerning the individuals’ autonomy and choice about diet, meal and snacking patterns; source of foods; and with whom meals are eaten.

#### Physical activity measurement

Physical activity will be objectively measured for 7 days at each time point using ActiGraph GT3X accelerometers (ActiGraph, Pensacola, FL). Participants will be shown by the researcher how to wear the monitor and will provide a wear instruction leaflet to take home to parents. Participants will be asked to wear the accelerometer for 7 days at baseline, 6 and 12 months, on the right hip, attached with a belt, during all waking hours, except when showering, bathing or swimming. The participants will offer a range of ‘tips’ to help them to remember to wear the accelerometer, e.g. hang it on the bedroom door or leave it on the pile of clothes to be worn the following day. Previous research by the authors indicates reasonable compliance of wearing the accelerometers for the wear time specified [37]. The monitors are small, lightweight and worn around the waist. Clothing is worn over the top of the monitor, minimising the visibility of the devices. The researcher will explain to participants that they are not to do any extra physical activity over and above their normal participation. The monitor does not come with any safety risks and will be activated by the researcher at the study visit. It will start recording data at this point so that participants will not be required to do anything to the monitor during the week that they wear it.

Accelerometry is a valid measure of physical activity [47] and has been widely used in young adults with intellectual disabilities [48]. A 7-day monitoring period also provides reliable (ICC = 0.76–0.86) estimates of physical activity, including weekday and weekend differences, in young adults [49]. The ActiGraph quantifies physical activity in counts/minute, which can be used to estimate time spent in sedentary, light, moderate and vigorous intensity activity and can enable the calculation of activity energy expenditure [50]. In keeping with guidelines on the validity of accelerometer data, the minimum data requirement will be set at 6 h of data on at least 3 days from seven, to ensure a valid measure of physical activity levels.

Each stage of data collection will consist of an average of two visits to each participant, roughly one week apart. At the second visit, the researcher will check the accelerometer wear time by plugging the accelerometer into a laptop and downloading the data through the actilife software. If the wear time does not meet the criteria (stated above), the researcher will invite the participant to wear the accelerometer for one more week. In such cases, a third visit will be made to collect the device.

#### Measuring self-determination

We have reviewed measures of self-determination used in studies involving individuals with intellectual disabilities. Most of these measures are designed to measure self-determination in relation to the school curriculum and are therefore not relevant to this study. The choice questionnaire [51] will be used in this feasibility study, although some of the wording will be adapted to ensure its relevance to younger participants living at home. The feasibility of measuring levels of self-determination using the choice questionnaire will be examined. This is a 26-item questionnaire designed to measure the degree of self-determination available to individuals with intellectual disabilities. The measure includes questions about home life, staff (family), money and spending, health, social activities, community access, personal relationships, work/day activities and overall self-determination. For example, can you get yourself a drink or something to eat whenever you want? Any time? Do you have to ask someone first? (a) Yes. I can have a drink or snack whenever I want. (b) I can usually have a drink or a snack but I have to ask first. (c) No. I am not usually allowed to have snacks and/or drinks OR I can only have them on special occasions. There are three options of ‘choice’ for each question. The interviewer will also follow up on any questions that are specifically relevant, to the study. For example, if the participant stated that he/she has to ask to have a snack, the researcher would enquire who they ask and what kind of snacks they like to have. This provides more context about each individual’s life and also gives an insight into potential environmental mediators (e.g. who is making decisions, why are certain decisions made) that may affect lifestyle behaviours. The scale has been shown to have sound reliability, validity, satisfactory test-retest reliability, internal consistency, interrater agreement, interscore agreement, content validity, concurrent validity and construct validity [51]. The researcher will read the questions aloud to participations and record responses on the questionnaire. The questionnaire should take 10–20 min to complete, depending on the amount of contextual detail provided. Overall, the questionnaires together will take around 45–60 min to complete (see Additional file 2).

To examine contextual information about individuals’ lives relevant to self-determination, individual interviews with participants in the nested study will explore feelings of autonomy related to lifestyle behaviours. Questions will be included about potential environmental mediators of self-determination identified in other studies [34, 35] to examine their relevance to transition, lifestyle behaviours and health.

### Semi-structured interviews

Ten participants will be selected for semi-structured scoping interviews. To ensure a range of pupils are included in the interviews, no more than three pupils will be invited from each participating school/club. If recruitment is successful from a small number of schools/clubs, the researcher will invite more pupils from each school/club. To ensure a range of experiences are captured, the researcher will select up to three participants that vary in their PE/sport participation and dietary behaviours. This information will be based on the questionnaire data which will be collected prior to the interview selection.

Interviews will be semi-structured and encompass the following areas: school and out-of-school sport and physical activity participation, activities liked/disliked in school PE and out of school, food eaten in and out of school, food preferences, choices about food and physical activity participation, and social and environmental influence on food and physical activity participation. During the interviews, activity cards will be used to talk about physical activity and diet (Additional files 3 and 4). These cards will contain images of different sport/exercise activities and a range of foods. The activity sheets have been designed primarily as a trigger to promote discussion around physical activity and diet and will also serve as a communication aid. For example, participants will be asked to sort activity cards into three piles: things they like, things they are unsure about and things they do not like doing/eating. This will facilitate interaction with pupils who struggle with verbal communication.

A longitudinal method [52] will be used, interviewing participants at baseline and 12 months to gain information about lifestyle behaviours and self-determination (see Additional file 5). This offers the opportunity to become familiar with the participants, allowing exploration and understanding of the young people’s school and home environment and the influences which may affect lifestyle behaviours. In addition, a longitudinal design will allow exploration of how, why and when changes in weight and self-determination occur and will provide contextual information about the environments in which they occur. This will provide insight into the importance of self-determination related to lifestyle behaviours and will also assess feasibility of measuring diet patterns, eating habits and food frequency, in this population. A systematic approach to data analysis will be adhered to when analysing the data. This includes recognising and describing patterns, themes and typologies across participants. Participants will also be invited to identify a family member to take part in a separate interview, which will be carried out at the 12-month data collection point. The interview data will be used to inform future outcome and intervention studies. Carer involvement has been shown to be important in lifestyle behaviour change for adults with intellectual disabilities [28, 53]. Interviews with family members will allow us to gain insight into the influence that family can have on lifestyle behaviours in young people with intellectual disabilities. In addition, understanding more about how this influence may or may not change over this transition will be important for future weight gain prevention research.

## Discussion

While there is a strong evidence base which suggests that the transition from school to adulthood is a high-risk period for weight gain in the general population, there is no current evidence to support this contention with the intellectual disability population. Furthermore, there are no published feasibility studies examining the lifestyle behaviours of young people with intellectual disabilities during their transition to adulthood. A vital first step is to find out if it is feasible to recruit and retain young people with intellectual disabilities, to a study examining their lifestyle behaviours. In addition, understanding more about self-determination and the context of young people’s lives will facilitate the development of this important body of work.

Evidence of effective recruitment and retention rates would provide a rationale for a future outcome study, to assess the changes in physical activity and diet during the transition to adulthood. Future work could compare the lifestyle transitions in young people with intellectual disabilities to typically developing young people. The outcome measures used in this study have been used previously in typically developing youth (with the exception of the choice questionnaire), therefore allowing for direct comparison in lifestyle behaviours over this transition in those with and without intellectual disabilities. Information gathered about the context of young people’s lives which may affect lifestyle behaviours will be instrumental in designing and developing an intervention to prevent weight gain. Following this feasibility study, the authors aim to develop an RCT to test the effectiveness of a weight gain prevention intervention. This trial would have two key aims: (1) to develop an intervention which is effective in preventing weight gain in young adults with intellectual disabilities over the 5-year transition to adulthood and (2) to increase levels of self-determination for lifestyle behaviours in individuals with intellectual disabilities. The RCT would compare an intervention group to a similar control group, consisting also of individuals with a mild-moderate intellectual disability. The overall aim of this work will be to tackle the rising rates of obesity, a significant health inequality faced by this population.

## Additional files

Additional file 1:
**Recruitement strategy framework.** (PDF 185 kb)
Additional file 2:
**Moving on and feeling good study.** (PDF 604 kb)
Additional file 3:
**Food activity cards.** (PDF 1227 kb)
Additional file 4:
**Physical activity cards.** (PDF 139 kb)
Additional file 5:
**Individual interviews with participants at school (baseline).** (PDF 334 kb)
