# Supplementary material for: ‘Moving on and feeling good’: a feasibility study to explore the lifestyle behaviours of young adults with intellectual disabilities as they transition from school to adulthood—a study protocol
Source: Pilot Feasibility Stud. 2016 Jan 29;2:8. doi: 10.1186/s40814-015-0044-9 (PMC5154056; doi:10.1186/s40814-015-0044-9)
Supplement: Additional file 1: — Recruitement strategy framework. (PDF 185 kb) [file 40814_2015_44_MOESM1_ESM.pdf]

Appendix 6 Recruitment strategy framework

Stage 1- pool

a) Identify target group within population or setting (November- December 2014)

Establish eligibility to take part:

Young people, over 16 years, in final year of school, mild to moderate intellectual disabilities and independent ambulatory.

b) Formative evaluation of recruitment approaches

A multi- point recruitment strategy will be used to recruit from three main sources. ASN schools will be targeted first, followed by mainstream schools and disability clubs:

- 1. Additional Support Need (ASN) schools
- 2. Mainstream schools
- 3. Disability clubs in Greater Glasgow.

Stage 2a- ASN Schools invited

a) Offer invitation (December 2014- June 2015)

The researcher will visit the interested ASN schools and speak to the teachers about the study. Information packs will be given out to teachers who interested in their school taking part in the research.

Stage 2b- pupils invited

The researcher will visit the interested ASN schools and speak to the pupils about the study.

Foster et al (2011) suggest that recruitment and retention to physical activity studies can be strengthened if participants are invited to participate face to face. Thus, the researcher will visit each interested participant in their school/club to provide more information about the study.

Information packs will be given out to pupils who interested in taking part in the research.

These will contain a letter introducing the study and information sheets for:

a) The participant,

b) A parent/guardian.

Parental consent is not required as all pupils over 16 years with mild-moderate LD will have capacity to consent. Parents will however be provided with information about the study. Staff will be asked to support individual read and understand the information pack, but importantly, they should not suggest how they respond. Pupils can indicate their interest to participate verbally, or the can complete the tear of form and give to their teacher. The teachers will then inform the researcher of any forms that have been handed in.

This will be explained to staff.

b) Monitor response uptake (January 2015-June 2015)

Consent forms can either be filled in while the researcher is present or these can be left with participants to allow them time to consider their participation.

As monitoring the responses allows the researchers to evaluate the effectiveness of the recruitment strategy (Foster et al, 2011), the researcher will monitor how many pupils have given consent to take part in the study and which school/club the participants were recruited from. This will allow the team to asses to most effective recruitment point.

Telephone reminders have been identified as an effective strategy for recruitment (Treweek et al, 2010), therefore, the researcher will phone the schools/clubs to remind of any consent forms that have not been completed and will arrange a day/time to collect these. If an information pack has been lost, the researcher will send out another information pack to the school, to be passed onto the parent or pupil.

Once 30 consent forms have been completed, the research team will review the number of consenting participants from each recruitment point and assess the success of the recruitment strategy.

If there are <30 participants recruited from ASN schools, the same strategy will be applied to mainstream schools and disability clubs, until the desired number of participants are recruited to the study (n ≥30).

Stage 3- responded (February 2015-June 2015) data collection begins

Participants who consent to the research will be visited by the researcher at their school/club. The researcher will agree a date/time with the school/club and pupil, to come and begin the data collection.
