# Supplementary material for: ‘Moving on and feeling good’: a feasibility study to explore the lifestyle behaviours of young adults with intellectual disabilities as they transition from school to adulthood—a study protocol
Source: Pilot Feasibility Stud. 2016 Jan 29;2:8. doi: 10.1186/s40814-015-0044-9 (PMC5154056; doi:10.1186/s40814-015-0044-9)
Supplement: Additional file 2: — Moving on and feeling good study. (PDF 604 kb) [file 40814_2015_44_MOESM2_ESM.pdf]

MOVING ON AND FEELING GOOD STUDY

Name ______________________________ School _______________________________

Age_______________ Participant ID _________________

Home Address ____

____

Phone number (home) ___________________________ Mobile _______________________

University of Glasgow

VIA VERITAS VITA

Hi,

Welcome to the moving on and feeling good study.

We would like to ask you some questions about the food you like to eat and the activities you like to do. Is it ok if we start with some measurements?

Anthropometric Measurements

**Height**	**___.___m**
	**___.___m**
**Weight**	**___.___kg**
	**___.___kg**
**Waist circumference**	**________cm**
	**________cm**
**Hip circumference**	**________cm**
	**________cm**

Dietary recall- The multiple pass method 24 hours.

1. List of foods eaten or drunk

Respondents are asked to report everything that they had to eat or drink (e.g. on the previous day between midnight and midnight) in an uninterrupted free flowing list.

Can you tell me what food you ate yesterday?

food	Full description (including brand)	Combinations	Quantity (e.g packaging, portion sizes)	Second helpings or leftovers	When was food eaten

Can you tell me what you drank yesterday?

drink	Full description (including brand)	Combinations	Quantity (e.g packaging, portion sizes)	Second helpings or leftovers	When was drank

3. A recall review

The interviewer reviews all of the food eaten and drunk in chronological order, prompting for any additional eating or drinking occasions and foods or drinks consumed, and clarifying any ambiguities regarding the type of food or drink consumed and portion size. Finally the interviewer asks the respondent to select a place name for each eating

DINE: Dietary Instrument for Nutrition Education

Do you follow a special diet of any kind?

If yes, what is this?

1. About how many **pieces or slices per day** do you eat of the following types of bread, rolls, or chapatis? (Choose one answer on each line)

**Breads & Rolls**	None	Less than 1 a day	1 to 2 a day	3 to 4 a day	5 or more a day
White bread or rolls	0	1	4	9	13
Brown or granary bread or rolls	0	2	7	15	22
Wholemeal bread or rolls	0	3	8	18	26

Bread	

2. About how many **servings per week** do you eat of the following types of breakfast cereal or porridge? (Choose one answer on each line)

**Breakfast cereals**	None	Less than 1 a week	1 to 2 a week	3 to 5 a week	6 or more a week
Sugared type: Frosties, Coco Pops, Ricicles, Sugar Puffs<br>Rice or Corn type: Corn Flakes, Rice Krispies, Special K	0	0	0	1	2
Porridge or Ready Brek<br>Wheat type: Shredded Wheat, Start, Weetabix, Fruit 'n Fibre, Puffed Wheat<br>Muesli type: Alpen, Jordans	0	1	2	5	7
Bran type: All-Bran, Bran Flakes, Country Bran	0	2	5	12	18

Cereal	

3. About how many **servings per week** do you eat of the following foods? (Choose one answer on each line)

**Vegetable foods**	None	Less than 1 a week	1 to 2 a week	3 to 5 a week	6 to 7 a week	8 to 11 a week	12 or more a week
Pasta or rice	0	0	1	3	4	6	8
Potatoes	0	0	1	3	5	8	10
Peas	1	1	3	8	12	16	24
Beans (baked, tinned, or dried) or lentils	1	1	4	10	15	20	30

Other vegetables (any type)	0	0	1	2	3	5	6
Fruit (fresh, frozen, or canned)	0	0	1	3	5	8	10

Vegetables	

**Fibre Rating**	Less than 30	=	Low Fibre Intake
	30 to 40	=	Medium Fibre Intake
	More than 40	=	High Fibre Intake

**Total**	

4. About how many **servings per week** do you eat of the following foods? (Choose one answer on each line)

	None	Less than 1 a week	1 to 2 a week	3 to 5 a week	6 or more a week
Cheese (any except cottage)	1	1	2	6	9
Beefburgers or sausages	1	1	2	4	6
Beef, pork, or lamb (for vegetarians: nuts)	1	1	2	6	9
Bacon, meat pie, processed meat	1	1	2	5	8
Chicken or turkey	0	0	1	3	5
Fish (NOT fried fish)	0	0	0	1	2
ANY fried food: fried fish, chips, cooked breakfast, samosas	1	1	2	6	9
Cakes, pies, puddings, pastries	1	1	2	5	8
Biscuits, chocolate, or crisps	1	1	2	4	6

Score	

5. About how much of the following types of milk do you yourself use **per day**, for example in cereal, tea, or coffee? (Choose one answer on each line)

**Milk**	None	Less than a quarter pint	About a quarter pint	About half a pint	1 pint or more
Full cream (blue top)	0	1	3	6	12
Semi-skimmed (green striped [illegible]	0	0	1	3	6
Skimmed (red checked top)	0	0	0	0	0

Milk	

6. About how many **rounded teaspoons per day** do you usually use of the following types of spreads, for example on bread, sandwiches, toast, potatoes, or vegetables? (Choose one answer on each line)

**Spreads**	None	1	2	3	4	5	6	7 or more

**Regular margarine or butter** or **Reduced fat spread** such as sunflower or olive spread, Flora, Vitalite, Clover, Olivio, Stork, Utterly Butterly	0	4	8	12	16	20	24	28
**Low fat spread** such as Flora Light, St. Ivel Gold, Half-fat butter, Olivite, Flora Pro-activ, Light spread	0	2	4	6	8	10	12	14

Spread	

**Fat Rating**	Less than 30	=	Low Fat Intake
	30 to 40	=	Medium Fat Intake
	More than 40	=	High Fat Intake

Total	

7. What type of fat do you usually use for the following purposes?
(Choose one answer on each line)

	**Butter, lard, or dripping**	**Solid cooking fat** (White Flora, Cookeen) **Half-fat butter** **Hard margarine** (Stork)	**Soft margarine** (sunflower, soya) **Reduced fat spread** (olive, Flora Buttery, Olivio)	**Vegetable oil** or **Low fat spread** (Flora Light, Olivite, St. Ivel Gold)	**No fat used**
On bread and vegetables	1	2	3	4	3
For frying	1	2	3	4	3
For baking or cooking	1	2	3	4	3

**Unsaturated Fat Rating**	Less than 6	=	Low Unsaturated Fat
	6 to 9	=	Medium Unsaturated Fat
	More than 9	=	High Unsaturated Fat

Total	

Choice questionnaire

Acquiescence and recency items must be "passed". They may be asked before or during the first part of the checklist.

RECENCY ITEMS:

a. Do you live by yourself or with other people? [ ] By myself [ ] With others

b, Do you live with other people or by yourself?. [ ] By myself [ ] With others

Actual situation:

Lives alone/with others

(Circle correct alternative)

ACQUIESCENCE ITEMS

Most mornings do **you** pick what clothes to wear?

[ ] Yes [ ] Sometimes [ ] No

Most mornings does **someone else tell** you what clothes to wear?

[ ] No [ ] Sometimes. [ ] Yes

I DOMESTIC ACTIVITIES, STAFF AND THE OTHER PEOPLE YOU LIVE WITH

I. Who decides what time you go to bed? (Does anyone **tell** you what time to go to bed? Are there any rules about what time you should go to bed?)

[ ] I decide for myself.

[ ] I usually decide with help. Sometimes others tell me.

[ ] I have a set bedtime. Or others mostly tell me when to go to bed.

2 Who decides which jobs **you** do around the house? Do you have set jobs or a jobs roster? Who works out the roster/set jobs?

[ ] I/we (the residents) choose the jobs I/we do.

[ ] I/we (the residents) usually choose with help from staff. OR I/we help staff make up the jobs roster.

[ ] Others mostly tell me. OR My jobs are set by a jobs roster or list made up by someone else.

3 When you cook dinner, who chooses what you cook? Do you ask the others who live here what they would like to eat?

[ ] I choose (I may check with other residents to see what they do/don't like).

[ ] I usually choose with help from staff.

[ ] I don't cook dinner (or I only help with cooking) OR Others mostly choose what I cook OR There is a planned menu made up by someone else.

4 Can you get yourself a drink or something to eat whenever you want? Any time? Do you have to ask someone first?

[ ] Yes. I can have a drink or snack whenever I want.

[ ] I can usually have a drink or a snack but I have to ask first.

[ ] No. I am not usually allowed to have snacks and/or drinks OR I can only have them on special occasions.

5 What rules are there about using the telephone? Can you ring up whenever you

want to?

[ ] I can ring up without restrictions whenever the phone is not being used.

[ ] I can usually ring up. I may ask staff first. There may be minor restrictions (e.g. can't talk for too long if others want to use the phone).

[ ] I am unable or not allowed to use the phone OR my telephone use is restricted (e.g. only allowed to ring at specified times or to certain places or limited to local calls only).

6 Who picks the staff to work in your house? (Do you interview new staff to decide who will get the job? Are you asked what you think about new staff?.)

[ ] I/we (the residents) are responsible for deciding which staff will be employed (e.g. I sit on interview panels).

[ ] I/we (the residents) participate in choosing staff- e.g. I am asked for my views about new staff.

[ ] Others choose the staff. I am not consulted and have no real say about who works in my house.

7 Are there any rules in your house? Who makes up the rules for your house? (Do not include rules imposed in the lease or by the landlord.)

[ ] There are no rules (except the landlord's rules in the lease). OR I/we (residents) decide the rules

[ ] I/we (the residents) decide the rules with help from others.

.[ ] Others decide the rules. I have no real say.

8 Do you have your own key to the house? Do staff have keys to your house? Did you give them the key? Who says which people can have a key?

[ ] I have a key. Staff do not have keys OR I/we (the residents) decide who can have a key. I have a fair say.

[ ] I have a key, but others mostly decide who else also has a key.

[ ] I do not have a key. I have no real say about who has a key.

9 Who decides if you can have a pet [like a dog, a bird or goldfish] if you want you have any kind of pet you want. Do you have to ask anyone before you get a pet?

[ ] I can have any pet I like with no restrictions.

[ ] There may be some restrictions (e.g. on the type of pet) because of my lease/landlord. OR I have to ask others first.

[ ] I am not allowed to have a pet. OR others decide and I have no real say.

10 What rules are there about you being by yourself in the house [by yourself and without staff]? Can you be by yourself in the house if you want to? Anytime?

If person lives alone score as 3(i.e. no restrictions).

[ ] I can be by myself in the house at any time with no restrictions.

[ ] Sometimes I can be by myself (e.g. only in certain situations or for short periods -

1 - 2 hours).

[ ] I am not allowed to be by myself. I never am by myself in the house.

I I MONEY AND SPENDING

11 Who works out your budget so you will have enough money?

[ ] I budget my own money without assistance.

[ ] I have help budgeting my money.

[ ] Others budget my money and I have little say. OR I have a fixed budget worked out by others.

12 Who decides **how much** money you take out of your bank account? Can you

take out as much as you want? Do you ask anyone how much to take out?

[ ] I decide without help and with no restrictions on how much to withdraw.

[ ] I have help to decide how much to take out OR I ask someone how much to take out OR I have a limit on how much I can withdraw.

[ ] Others mostly decide how much to withdraw and I am not consulted.

13 When you buy your clothes who chooses which clothes to buy?

[ ] I choose. I buy my clothes with no help.

[ ] I usually choose my clothes with help (e.g. someone usually goes with me).

[ ] Others mostly decide OR others buy clothes for me.

14 Do you spend some money on gambling like lottery tickets, lotto, poker machines or the TAB? Who decides that you do/don't gamble? (Can you gamble if you want to?)

[ ] I decide.

[ ] I usually decide with help. OR Sometimes others may tell me not to.

[ ] I am not allowed to gamble.

III HEALTH

15 Does anyone go with you to see the doctor and the dentist? Who? Does ..... always go?

[ ] I always go by myself or with a friend.

[ ] staff or family (e.g parents) come with me to some appointments (e.g. specialists)

[ ] I (almost) always go with staff or family (e.g. parents).

16 Do you drink alcohol like beer or wine? Who decided that you do/don't drink beer/wine? Do you ask anyone if you can drink alcohol? Who? *(If person drinks:* Does anyone try to stop you drinking alcohol? *If person does not drink:* Why is that?)

[ ] I decided. I am free to drink or not.

[ ] I decided with help OR I ask someone (staff or family) first OR I don't drink because of the medication I take or other medical reasons OR I drink but there are some restrictions on my drinking.

[ ] Others decide (e.g. say I am not allowed to drink).

17 Do you do exercise or play sport? Who decides that? (Does anyone make you

do exercise or sport?)

[ ] I decide.

[ ] I usually decide with help.

[ ] Others mostly decide. OR I am made to do exercise/sport.

IV **SOCIAL ACTIVITIES, COMMUNITY ACCESS AND PERSONAL RELATIONSHIPS**

18 Does anyone stop you from going out? Is there anywhere you are not allowed to go?

[ ] No-one stops me. I can go wherever I want.

[ ] There are 1 or 2 places I am told not to go.

[ ] Others often stop me going out. Or I am not allowed to go to quite a few places.

19 Who decides what you do in your spare time [when you are not at school/work or at day activities]?

[ ] I decide.

[ ] I usually decide with help.

[ ] Others mostly decide.

20 Who decides if you can go to hotels and clubs? (Does anyone try to stop you?)

[ ] I decide.

[ ] I usually decide with help.

[ ] Others mostly decide. OR I am not allowed to.

21 Who decides if you can go and visit your family and friends [whenever it is all right with them]? Do you ask anyone first? Who?

[ ] I can visit whenever it's okay with my family or friends.

[ ] I can visit but I ask someone (other than the person I am visiting) first.

[ ] Others decide. OR I am not allowed to visit.

22 Does anyone stop you from looking at sexy [X rated] magazines, videos or movies? (If the person says "I *don't look at those things",* ask: Who decided that?)

[ ] No. I can look at anything I want (in private). OR I decide not to.

[ ] Usually no-one stops me. Occasionally they may ask me not to. OR I decided with help not to.

[ ] I am not allowed to. OR I am only allowed to look at things some else says are okay.

V SCHOOL/WORK/DAY ACTIVITIES

23 Can you leave your School/job/day activity if you want to, do no work and just stay at home? Would you ask anyone first? (Would anyone try to stop you leaving if you wanted to?)

[ ] Yes, I can leave if want to. I don't have to ask anyone else. OR I have already left my school/job/activity & it was completely my decision.

[ ] I participate in the decision and discuss it with others.

[ ] Others decide OR I am not allowed to leave OR I have never had a job or day activities.

24 Can you be late home from school/work/day activities? Do you have to tell anyone first or ring up? (Do you get into trouble for being home late?)

[ ] I can come home when-ever I like. I don’t have to tell anyone first or ring up.

[ ] I can be late if I want, but I am supposed to ask/tell someone first or ring up. I get into trouble if I don't tell someone or ring up.

[ ] Others decide. OR I am not allowed to be late. OR I have no opportunity to stop off after work & get home late because I never go to school/work/day activities or because I am driven straight home.

25 What happens if you want to take a day off school/work/day activities when you are not sick? You just feel like having a day off. Do you have to ask anyone first?

[ ] It is my decision. (I might lose a day's pay).

[ ] I decide with help. I ask others (e.g. staff or family) first.

[ ] Others decide - I have no real say. OR I am not allowed. I have to go to school/work/day activities OR I do not make this choice because I never work or attend day activities.

VI OVERALL CHOICE

26 Overall would you say that your life is free so you can choose what you want?

All the time?

[ ] Yes, most of the time. Sometimes it is planned for me.

[ ] No. I often cannot do what I want.
