# Supplementary figures and images for: ‘Moving on and feeling good’: a feasibility study to explore the lifestyle behaviours of young adults with intellectual disabilities as they transition from school to adulthood—a study protocol
Source: Pilot Feasibility Stud. 2016 Jan 29;2:8. doi: 10.1186/s40814-015-0044-9 (PMC5154056; doi:10.1186/s40814-015-0044-9)

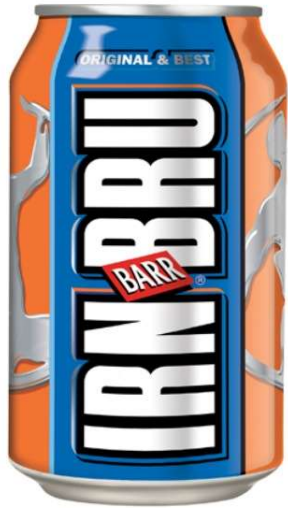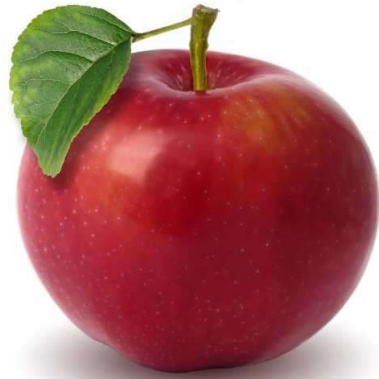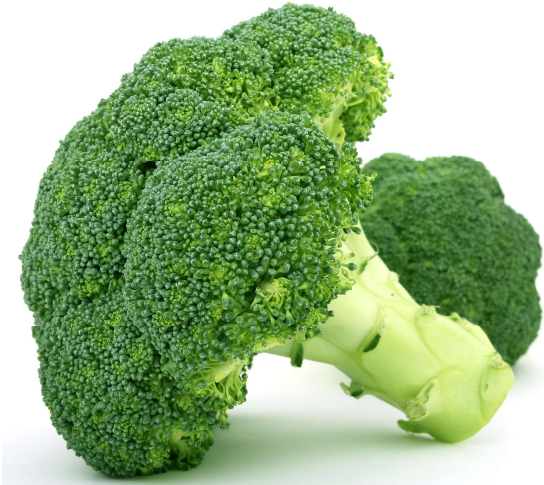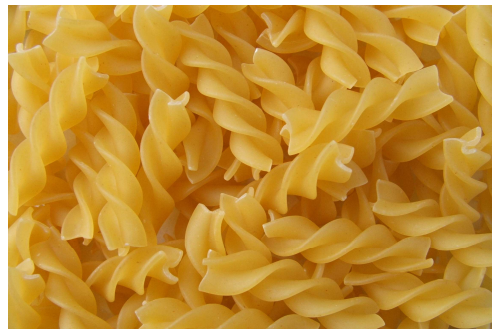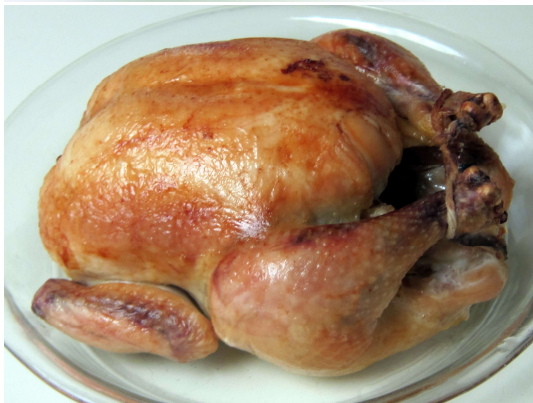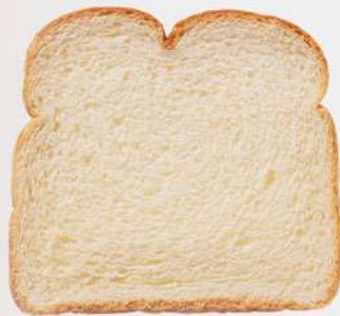

Supplement: Additional file 3: — Food activity cards. (PDF 1227 kb) [file 40814_2015_44_MOESM3_ESM.pdf]

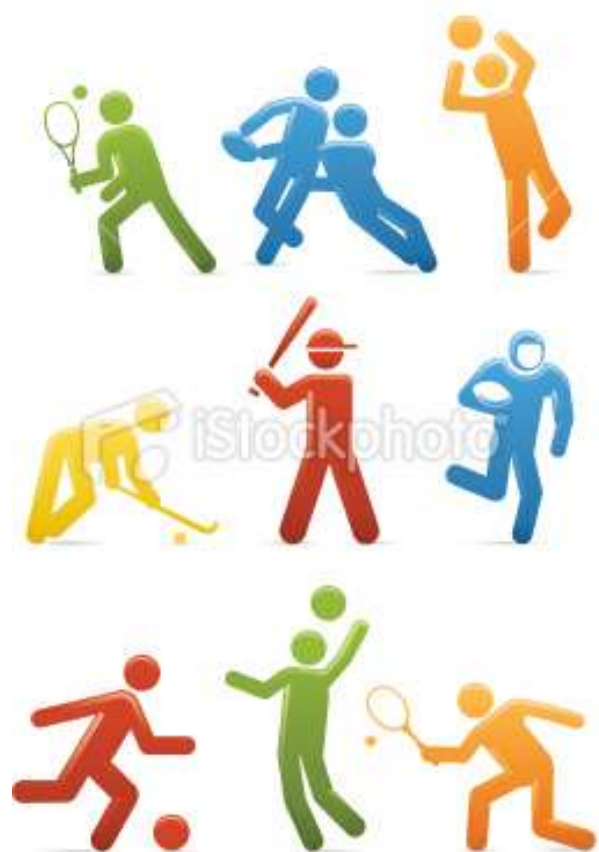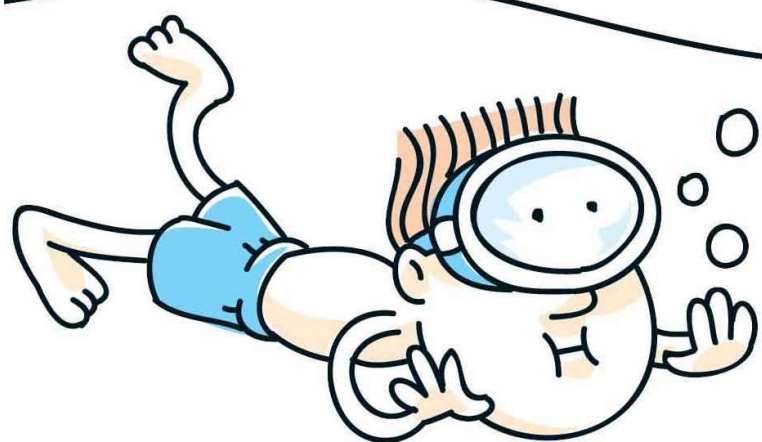

Supplement: Additional file 4: — Physical activity cards. (PDF 139 kb) [file 40814_2015_44_MOESM4_ESM.pdf]
