# Supplementary material for: ‘Moving on and feeling good’: a feasibility study to explore the lifestyle behaviours of young adults with intellectual disabilities as they transition from school to adulthood—a study protocol
Source: Pilot Feasibility Stud. 2016 Jan 29;2:8. doi: 10.1186/s40814-015-0044-9 (PMC5154056; doi:10.1186/s40814-015-0044-9)
Supplement: Additional file 5: — Individual interviews with participants at school (baseline). (PDF 334 kb) [file 40814_2015_44_MOESM5_ESM.pdf]

Individual interviews with participants at school (baseline)

The individual interviews will provide contextual information concerning the individuals' autonomy and choice about diet, meal and snacking patterns, source of foods, and with whom meals are eaten. This will allow us to understand more about the importance of self-determination related to lifestyle behaviours and will also allow us to pilot the feasibility of measuring diet patterns, eating habits and food frequency, in this population.

Introduction to interview

You have been taking part in the Moving On and Feeling Good project. I have already asked you a few questions about food and the activities you like to do. I have a few more questions I would like to ask you today, if that's ok? Are you happy to chat to me about this?

Great, so there are no right or wrong answers to these questions. I will not tell anyone what you have said and we won't mention your name when we write a report about the project. If you aren't sure what to say or don't want to answer some of the questions, that's fine. If at any time you want to stop just let me know. We will also do a couple of fun activities together.

If it's OK with you, I would like to use a tape recorder. This is just so I don't have to write everything down as you speak and I can listen properly to what you are saying. The tape will only be listened to by the person typing it up – no one else will hear what has been said. Your name won't be written anywhere. Can I ask if it is ok with you for me to use a tape recorder? If not, I will just make notes. Before we begin, do you have any questions you want to ask?

Individual and social factors

Part 1. School activity/sport preferences

I have something we can do together which can help you to think about what activities you like doing and what you don't like doing when you are at school.

1. Here are 6 pictures of people doing different things in *[the images include; badminton, football, dancing, basketball/netball, swimming, gymnastics*]. We can make three piles

    a) Is for activities you really like doing in school (point to space on the floor)
    b) Is for activities you don't mind doing/ or sometimes like doing in school.
    c) Is for activities you really don't like doing in school.

<u>*Have a blank card(s) to put other activities onto – draw or just write activities that you like/don't like.*</u>

Can you put each activity card into one of the three piles?

Go through each activity:

Can you tell me about the last time you did (the activity)?
Prompts- Who did you do (the activity) with? Where do you do (the activity)?

Who chose to do this activity?

Why did you put this activity into the ….. pile?

2. What activity do you have in PE just now? What do you think of it?

3. Are there any other activities/sport/games that you do in school that you really like?
Why do you like this activity?

4. Are there any other activities that you do in school that you really don't like?
Why don't you like this activity?

5. Do you do any activities/sports outside of school (after school or at weekends?)
If yes- Where do you do (the activity) ?
How do you get to ……….(activity/sport)? (prompt- does someone take you).
Does anyone take part in the activity with you? If yes, does ….. help you to do the activities/sport/games?
What do they do to help you?

Part 2: Food preferences

6. Now we are going to look at some pictures of different foods. We can do this activity together.

Here are some pictures of different food types.
We can make three piles again.

a) Is for food you really like eating (point to space on the floor)
b) Is for food you don't mind eating/or sometimes like eating.
c) Is for food you really don't like eating.

Have a blank card(s) to put other food onto – draw or just write activities that you like/don't like.

Go through each food eaten

7. Tell me about the last time you had (the food)?

8. Who do you eat this type of food with (e.g friends)?

9. Do you eat this food at school/home or both?

Meals

How many meals do you eat in one day?

Do you eat your meals at the same time every day?
Prompt what time for each meal?

Now, we will use the food mat and chose the foods we normally have for each meal.

Food eaten at school

What did you have for lunch at school today (or yesterday if early)

Where did you get lunch from?

Why did you have this for lunch?

Do you eat with anyone at school? If yes- who.

Have you had any other food/drinks at school today (e.g snacks)?
If yes- what have you had?

Did you buy the snacks?

When do you have snacks?

Do you like snacks you have? [would you prefer something different?]

What would your mum/dad think about you eating this food at school?

What do your friends think about you eating this food at school?

What do your teachers think about you eating this food at school?

Food eaten at home

Breakfast

Do you normally eat breakfast?

Where do you eat breakfast?

What do you normally eat for breakfast?

Who makes it? [if bought where from?]

Who decides what you have?

Why do you/they choose this?

Do you like the food you have for breakfast?

Do you eat breakfast with anyone? who

Lunch at home (weekends)

Do you normally eat lunch at the weekends?

What do you normally eat for lunch? (at weekends)

Who makes it? [if bought where from?]

Who decides what you have for lunch at the weekend?

Why do you/they choose this?

Do you like the food you have for lunch at the weekend?

Where do you eat lunch at the weekend?

Do you eat lunch with anyone at the weekend? Who?

Dinner

Do you normally eat dinner?

What do you normally have for dinner?

Who makes it? [if bought, where from?]

Who chooses what you have for dinner?

Why do you/they choose this?

Do you like the food you have for dinner?

Where do you eat dinner?

Do you eat dinner with anyone? Who?

Snacks

Do you have any snack during the day on weekends?

What snacks do you have?

Who makes/buys the snacks?

When do you have snacks?

Who chooses what you have?

Why do they/you choose this?

Do you like snacks you have? [would you prefer something different?]

Do you think you eat more food when you are at school or at home (or weekends and evening)? Why do you think this is?

Drinks

What do you drink when you are at school?

What do you drink when you are at home?

Who choses what drinks you have?

Why do you/they choose this?

Do you like the drinks you have? (would you prefer something different?)

Do you think you drink more drinks when you are at school or at home?

Knowledge/Views about health (environmental/policy level)

Do you know how much activity young people should do?

What do you think a healthy person is like?

What kind of things does a healthy person eat?

Do you think you are a healthy person? Why?

What do you think an un- healthy person is like?

What kind of things does an un- healthy person eat?

Do you think you are an un- healthy person? Why?

Do you think you need to make any changes to make you healthy/keep you healthy? What would they be?
